# Diagnosis of bone abnormalities in CKD-MBD (Imaging and bone biopsy)

**DOI:** 10.1590/2175-8239-JBN-2021-S103

**Published:** 2021-12-03

**Authors:** Sérgio Gardano Elias Bucharles, Lillian Pires de Freitas do Carmo, Aluízio Barbosa Carvalho, Vanda Jorgetti

**Affiliations:** 1 Universidade Federal do Paraná, Hospital de Clínicas Complex, Service of Nephrology, Curitiba, PR, Brazil.; 2 Universidade Federal de Minas Gerais, Belo Horizonte, MG, Brazil.; 3 Universidade Federal de São Paulo, Nephrology Discipline, São Paulo, SP, Brazil.; 4 Universidade de São Paulo, Pathophysiology Laboratory (LIM-16), Hospital das Clínicas da Faculdade de Medicina da USP, São Paulo, SP, Brazil.

## Recommendations on bone densitometry (DXA)

1. For CKD patients in all its stages and after kidney transplant (kidney Tx), the criteria for diagnosis of osteopenia and osteoporosis are the same as for the general population (Evidence). 

2. For patients with CKD G1-2, the same assessment routine with DXA as for the general population is suggested (Evidence).

3. For patients with CKD G3a-5D with the presence of CKD-MBD changes and risk factors for osteoporosis, bone densitometry (DXA) is suggested for fracture risk assessment (Opinion).

4. For patients with CKD G3a-5D with osteopenia or normal result by DXA, it is recommended performing the exam every 2 years (Opinion).

5. For patients with CKD G3a-5D with osteoporosis and/or fragility fractures, receiving antiresorptive treatment or treatment with anabolic agents, it is suggested performing a DXA examination every 1 year (Opinion).

6. For kidney transplant patients, it is recommended assessing fracture risk by DXA in the first six months after Kidney Tx (Opinion).

7. For stage Tx1-5 kidney transplant patients with risk factors for osteoporosis, it is suggested assessing fracture risk by DXA as frequently and in the same manner as for CKD patients (Opinion). 

## Rational

**Figure 1.**
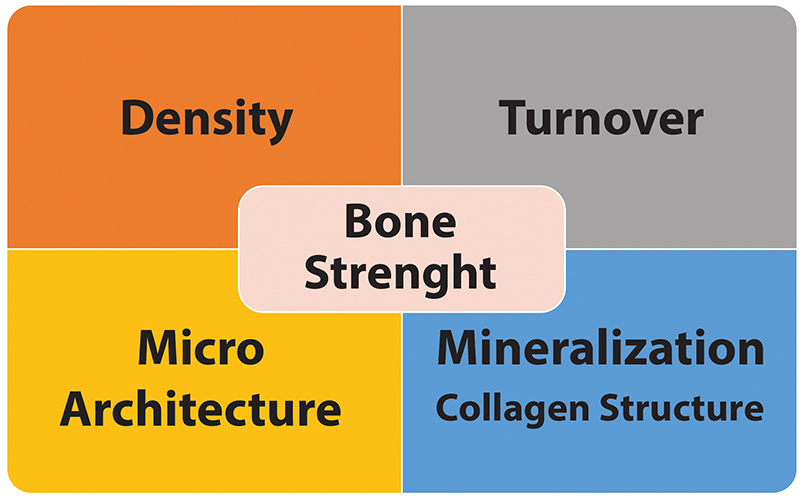
Components of bone strength

Renal osteodystrophy (RO) is the term used to describe the bone changes that occur during the course of CKD^1^. These changes impair turnover, mineralization, as well as cortical and trabecular bone microarchitecture, increasing the risk of fracture by reducing both bone mass and quality^1^ ^,^ ^2^. Bone strength (Figure 1) is defined by the characteristics of bone mineral density and bone quality. While bone mass (bone mineral density) could be assessed by two-dimensional radiological examinations (dual-energy X-ray absorptiometry - bone densitometry, DXA), bone quality, which refers to structural properties, includes turnover, microarchitecture, collagen arrangement, and mineralization aspects, and could not be adequately determined by DXA, requiring, when possible, assessment by other investigative radiological methods and even the use of bone biopsy itself^3^. 

Fractures are 2-14 times more frequent among CKD patients when compared to general population^4^ ^,^ ^5^. Their incidence and prevalence significantly increase as eGFR decreases and they are associated to high costs and morbidity and mortality^6^ ^,^ ^7^. The most recent update of KDIGO guideline for CKD-MBD^8^, when compared to previous guidelines, presented a change in the so far existing paradigm, henceforth recognizing the usefulness of radiological assessment, in particular by DXA, as an important discriminatory tool for fracture risk in the CKD population, based, among other information, on a compilation of specific clinical studies published in 2015^9^. We will briefly review recommendations for the use of radiological examinations in bone assessment of CKD, as well as the usefulness of FRAX tool (Fracture Risk Assessment Tool) in the CKD setting. 

## Bone Densitometry (DXA)

Considering its wide availability, low radiation exposure and more affordable cost, DXA is currently the most widely used tool in clinical assessment of bone mass and fracture risk, both in general population^10^ and in CKD setting^3^ ^,^ ^8^ ^,^ ^11^. Similar to what is postulated for the general population, T score values ≤ - 2.5 SD of normal are highly predictive of fracture risk among CKD patients^12^. A low bone mass detected by DXA in the distal third of the radius, femoral neck and lumbar spine is a predictor of fracture risk in patients with CKD G3-5D^8^. For children, premenopausal women under 40, and men under 50, the Z-score, rather than the T-score, should be used to assess bone mineral density (Z-score <-2.0)D^13^. 

Although the use of DXA as a predictor of fracture risk in CKD has historically been controversial, the most recent reviews on this topic have identified at least four prospective cohort studies using this tool, and studying the incidence of fragility fractures in patients with CKD G3-5D^2^ ^,^ ^8^ ^,^ ^9^. These studies have shown that bone mineral density (BMD) assessed by DXA was a predictive tool for fracture risk in CKD patients (Table 1)^12^ ^,^ ^14^ ^-^ ^16^, information that also seems to be valid for kidney transplant patients, particularly when there is osteopenia (T score from - 1.0 to - 2.4 SD) and BMD < 0.9 g/cm^2^ at the femoral neck^17^. These studies have also indicated that the same T-score values validated by the World Health Organization for diagnosing osteopenia and osteoporosis (OP) in the general population could be used for CKD patients^1^ ^,^ ^11^. 

**Table 1.** Major studies on CKD showing DXA as a predictor of fragility fracture risk

Main author	Year of publication	Population studied	Main findings
Iimori, S. et al.^14^	2012	CKD G5D in HD N = 485 Japan - single center Median age: 60 years old	↓ Baseline MBD (femoral head and total hip) predictor of fractures PTH > 204 pg/mL and ↑ BAP both biochemical predictors of fractures
Yenchek, R.H. et al.^16^	2012	CKD G3a-3b N = 587 US Population	For each 1 SD reduction in DXA, 2.6 x greater risk of fracture in CKD cases, both for femoral neck and total hip
West, S.L. et al.^12^	2015	Prospective cohort CKD G3a, G3b, G4 and G5 not on dialysis N = 131 Canadian population	For each 1 SD reduction in DXA there was a 1.75 x greater risk of fracture Low MBD at all sites was a predictor of fracture risk
Naylor, K.L. et al.^15^	2015	CKD G3a and G3b N = 320 Canadian population FRAX with or without DXA	FRAX with DXA, without DXA, and femoral head T score; all were predictors of fracture risk

Source: Adapted from reference 8.

In CKD patients who suffer fragility fractures, the main clinical dilemma is the differentiation between OP and the various presentations of RO (osteitis fibrosa, low-turnover bone disease, mixed bone disease, osteomalacia). This problem could be exacerbated insofar as RO and OP coexist, a more prevalent scenario in cases of advanced CKD^1^ ^,^ ^11^. Additionally, the same patient may present at different times with different RO patterns and, in the CKD setting, increased PTH could be anabolic for trabecular bone, but catabolic for the cortical one. Since DXA cannot separate these two components (RO and OP), its role in assessing bone strength is limited. It is also important to highlight that DXA, as it does not assess bone quality or the type of underlying RO, may be less predictive or underestimate fracture risk in patients with CKD G4-5D compared to earlier stages of CKD^1^ ^,^ ^18^. 

As a general recommendation, patients should undergo DXA in at least two distinct sites (femoral neck, lumbar spine or distal third of the radius), and the lumbar spine should be invalidated in cases of extensive vascular calcification or significant osteoarthritis. The optimal time interval for performing the exam is not known, but national guidelines for the treatment of OP suggest, in the case of patients at high risk for fractures, especially if they are receiving pharmacological treatment, to perform DXA every 1-2 years^9^. Furthermore, several studies suggest that the applicability of DXA may be enhanced by concomitantly performing fracture risk estimation by the FRAX tool (10-year fracture risk assessment) in kidney transplant patients and in CKD G3-G4^20^ ^,^ ^21^, with likely less utility of FRAX among CKD patients on renal replacement therapy (hemodialysis)^14^.

It is fundamental to note that the usefulness of DXA is primarily dependent on the quality of the images obtained, as well as their correct analysis and interpretation, based on well-established standardizations, in order to minimize errors of execution^22^ ^,^ ^23^. When serial exams of the same patient are performed, it should also be considered that there is a minimal significant variation. This corresponds to the intrinsic technical variability of the exam, calculated for each set consisting of the device used and the operating technician^22^. This process of certifying the exam quality consists of quantifying the bone density value twice consecutively in a set of thirty patients, or three times in a set of fifteen patients, with repositioning between the exams^22^ ^,^ ^23^.

## Trabecular bone score (TBS)

The two-dimensional nature of the spatial resolution of images obtained by DXA does not allow a direct assessment of bone microarchitecture (cortical thickness and trabecular volume)^10^. In order to add information in this sense, a computer program was developed to extract the DXA images, obtained from the lumbar spine, to evaluate the trabecular microstructure. Using a scale with different tones of gray, their homogeneity is evaluated, and the ratio is directly proportional to the quality of the trabecular structure organization^10^ ^,^ ^18^. Several radiology research centers have now incorporated TBS into the usual performance of DXA^18^. 

In prospective studies with a large numbers of patients, reduced TBS has been shown to be a good marker for fragility fracture risk in general population, regardless of DXA values and other major risk factors such as advanced age and previous fractures^24^ ^,^ ^25^. In the CKD setting, there is growing information regarding the usefulness of TBS. Naylor et al. conducted a multicenter study in patients with eGFR < 60 mL/min/1.73 m^2^ in the Canadian population and the results showed association of TBS with fragility fracture risk. Patients > 40 years old, with CKD, followed for 5 years, when compared to the population with normal renal function, had lower mean TBS (1,275 x 1,297) and a higher probability of fragility fractures among those with TBS values below the median obtained in the study^26^. 

In HD patients, Yavropoulou et al. observed, in a case-control study, that the 50 patients studied had significantly lower TBS values than the control group, a difference that remained significant after adjustments for age and PTH, 25OHD_3_, phosphorus and alkaline phosphatase values^27^. Brunerova et al., also investigating patients under HD, observed that half of their series (N = 59) presented severe alteration of the trabecular microarchitecture assessed by TBS, and that these findings correlated with the results of high-resolution peripheral quantitative computed tomography (HR-pQCT)^28^. More recently, Dusceac et al., studying 98 patients on HD, observed that, when compared to healthy controls, the patients had lumbar spine TBS values significantly lower and a 5-fold increased risk of fragility fractures^29^. 

Similarly, in the kidney transplant population, Naylor et al. observed that TBS values are significantly lower when compared to controls, and TBS was associated with higher fracture risk, again, regardless of FRAX and DXA^30^. Additionally, Perez-Saez et al. investigated the TBS in a population of long-term kidney transplant patients (mean follow-up of 10 years) and noticed that TBS values on average were lower when compared to healthy controls, findings independent of DXA values and corticosteroid use^31^. Luckman et al. studied longitudinally 47 kidney transplant recipients for 12 months, assessed with DXA, TBS and HR-pQCT. At one year follow-up, only 50% of patients had TBS values as being at low risk for fragility fractures (>1,370), and 42% of the population, although presenting with DXA within normal range, were classified as at high risk for fractures, based on TBS values. Furthermore, TBS values correlated significantly with HR-pQCT in trabecular thickness and bone density parameters^32^. 

Altogether, these studies highlight that there is significant damage to bone microarchitecture assessed by TBS, confirming its role as a predictor of fragility fracture risk in the population with CKD G3a-G5D and in kidney transplant recipients, making it reasonable to suggest that, when available, this tool should be used as a predictor of fracture risk in this population^33^.

Finally, some considerations with respect to HR-pQCT, which has the advantage of presenting a resolution of 60-82 µm^3^, providing detailed information in three dimensions regarding bone microarchitecture and its geometry, quantifying and qualifying the trabecular bone (thickness and number of trabeculae), as well as assessing cortical porosity^1^ ^,^ ^34^. This modality of investigation does not assess bone turnover and mineralization and thus may not provide information regarding the type of RO of the evaluated patient^1^. Cross-sectional studies performed in patients with CKD G3a-5D demonstrated that the HR-pQCT parameters assessed in tibia and distal radius were associated with fragility fractures^35^ ^,^ ^36^.

Although this exam has validated applicability in the CKD setting, its limited availability and higher cost determine that it is not recommended as a routine exam for detecting OP and assessing fracture risk in CKD^34^. Table 2 presents the advantages, disadvantages and perspectives related to the use of different methods of radiological investigation in CKD-MBD. 

**Table 2.** Advantages, limitations and perspectives of the methods for radiological assessment of bone tissue in CKD-MBD

Method	Advantages	Disadvantages	Perspectives
DXA and TBS	Non-invasive Low cost Accessible Predictor of fracture risk Reports trabecular microarchitecture (TBS)	No information on bone turnover and mineralization No differentiation between cortical and trabecular bone (DXA)	Use in intervention studies in the immediate future
HR-pQCT	Non-invasive High Definition High sensitivity Differentiation between cortical and trabecular bone	Not widely available High Cost No information on bone turnover and mineralization Still not consistent as a predictor of fracture risk	Greater availability in the near future Future prospective studies for definition as a fracture risk tool
Bone biopsy	High definition and sensitivity ("gold" standard) Differentiation between cortical and trabecular bone Presenting information on bone turnover and mineralization	Invasive High Cost It requires expertise Not assessed as a predictor of fracture risk	Growing interest in the method - new research groups Future studies integrating radiological methods and bone biopsy

DXA: Bone densitometry; TBS: Trabecular bone score; pQCT: Peripheral Quantitative Computed Tomography

Source: Adapted from reference 33.

## Role of FRAX (Fracture Risk Assessment Tool)

In the general population, the use of FRAX as a discriminating tool for fracture risk is widely accepted and incorporated in several guidelines for the assessment and treatment of OP^37^. The instrument relies on the analysis of eleven clinical variables and optional additional information from DXA obtained at the level of the femoral neck, but not including the presence of kidney disease. Thus, although FRAX does not include adjustments for eGFR, it is suggested that the tool is useful as an initial assessment for both CKD and Kidney Tx patients, although probably underestimating the real risk of fracture^38^. 

Jamal et al. observed in CKD patients that the fracture risk discriminating ability of the DXA at the femoral neck was similar to FRAX for morphometric vertebral fractures, with FRAX being of superior utility for non-vertebral fractures^21^. Naylor et al. studied, using FRAX and DXA, 320 patients with eGFR < 60 mL/min/1.73m^2^ and 1,787 patients with eGFR > 60 mL/min/1.73m^2^ ^,^ ^15^. For patients with CKD, the observed risk of fragility fracture was 5.3%, comparable to the FRAX estimate (6.4% with DXA and 8.2% without DXA)^15^. Additionally, Whitlock et al. studied a cohort of over 10,000 patients, including 2,154 patients with CKD G3a and 3b and 590 patients with CKD G4-G5. During a mean follow-up of five years, it was observed that for each increase in standard deviation of FRAX values, the risk of fragility fracture was significantly higher and adequately captured by FRAX, with or without the use of DXA, in all stages of CKD^39^. Furthermore, Przedlacki et al., studying 718 patients with CKD 5D (on HD), noticed that, in logistic regression analysis, FRAX was the most robust independent factor in assessing fracture risk in that population^40^. Finally, among 458 kidney transplant patients, Naylor et al. concluded that the observed 10-year risk of fracture was 6.3%, similar to the values stipulated by FRAX (5% with DXA and 5.6% without DXA)^20^.

Despite all these results, further studies are needed before FRAX could be more widely recommended in daily practice, in particular for patients with CKD G4-5D, since the presence of CKD-MBD in this population more significantly affects bone metabolism and carries with it particular treatment implications (vitamin D analogues, calcimimetics, phosphate binders), potentially interfering with fracture risk assessment and subsequent treatment.^38^

### Recommendations on bone biopsy

1. Tetracycline double labeling bone biopsy followed by histomorphometric analysis is the gold standard for diagnosis and classification of renal osteodystrophy (RO) (Evidence).

2. In patients with CKD G3a-5D, bone biopsy should be considered in the following conditions: fragility fractures, refractory and unexplained hypophosphatemia and/or hypercalcemia, suspected aluminum toxicity, discrepancy between serum biomarkers and clinical presentation; and before starting anti-osteoporotic drugs (Opinion).

### Rational

Renal osteodystrophy (RO) is defined as the set of changes in bone histology that are part of the spectrum of manifestations of mineral and bone disorder in chronic kidney disease (CKD-MBD) ^41^. Bone biopsy with tetracycline double labeling, followed by histomorphometric analysis, is the gold standard for diagnosis and classification of renal osteodystrophy, as it is the only method capable of providing the assessment, in trabecular and cortical bone, of structural and dynamic parameters of bone histology^42^ ^,^ ^43^. Bone biopsy therefore provides information on volume (V), turnover (T) and mineralization (M), which serve as a basis for classifying the type of RO^44^.

The RO treatment depends on the type of bone alteration found, whether high or low turnover, which presumptive diagnosis through the measurement of serum biomarkers is not always accurate^45^ ^,^ ^46^ . Nonetheless, as outlined in other chapters of this guideline, we reinforce the importance of assessing the tendency of PTH and alkaline phosphatase levels to guide therapy^45^. Non-invasive methods, as for example bone densitometry, quantitative computed tomography and magnetic microresonance imaging, although capable of assessing bone mass and microarchitecture, do not assess turnover or mineralization, nor do they determine the type of RO.

As an invasive method that requires specialized centers to perform it, bone biopsy is not recommended as part of routine assessment in CKD^43^. We suggest that in patients with CKD stage 3 to 5D, bone biopsy should be considered mainly in the following conditions: (i) fragility fractures; (ii) refractory, unexplained hypophosphatemia and/or hypercalcemia; (iii) suspected aluminum toxicity, if the desferrioxamine test is inconclusive or could not be performed; (iv) discrepancy between serum biomarkers and clinical presentation; (v) before starting anti-osteoporotic drugs. However, although biopsy could provide important information to guide the osteoporosis therapy, its performance is not mandatory, and the impossibility of performing it should not be considered an impediment to initiating osteoporosis treatment, particularly in patients with CKD G3a, 3b and 4, for whom the antiresorptive treatment has been shown to be safe and effective^47^. The aims of bone biopsy are to discard atypical or unexplained disease by clinical presentation and biomarkers, to determine whether the patient has high or low turnover disease that may alter the treatment (such as starting or discontinuing calcimimetics or vitamin D analogues), or to identify mineralization defects that need specific treatments ^47^.

Tetracycline labeling of bone tissue prior to bone biopsy is important to allow proper histomorphometric assessment^43^. A detailed description on how to perform tetracycline double labeling, the biopsy procedure, its care, and complications, are beyond the scope of this chapter and can be found in other publications^42^ ^,^ ^43^.

The expansion of the therapeutic arsenal for treatment of CKD-MBD and osteoporosis may eventually require the use of bone biopsy to enable a more individualized treatment, which is not always possible only through clinical presentation and the use of serum biomarkers^48^. This reinforces the importance of a greater number of nephrologists becoming qualified to perform the procedure and histomorphometric analysis of bone tissue.
